# Identification of Short-Chain Fatty Acids for Predicting Preterm Birth in Cervicovaginal Fluid Using Mass Spectrometry

**DOI:** 10.3390/ijms25063396

**Published:** 2024-03-17

**Authors:** Young-Min Hur, Eun-Jin Kwon, Young-Ah You, Sunwha Park, Soo-Min Kim, Gain Lee, Yoon-Young Go, Young-Ju Kim

**Affiliations:** 1 Department of Obstetrics and Gynecology, Ewha Medical Research Institute, College of Medicine, Ewha Womans University, Seoul 07984, Republic of Korea; hym1210@ewha.ac.kr (Y.-M.H.); yayou@ewha.ac.kr (Y.-A.Y.); clarrissa15@gmail.com (S.P.); zeus_0218@ewhain.net (S.-M.K.); loveleee0102@gmail.com (G.L.); gokogoko@ewha.ac.kr (Y.-Y.G.); 2 Division of Allergy and Respiratory Disease Research, Department of Chronic Disease Convergence Research, Korea National Institute of Health, Cheongju-si 28159, Republic of Korea; friendkej1004@hanmail.net

**Keywords:** preterm birth (PTB), short-chain fatty acid (SCFA), cervicovaginal fluid (CVF)

## Abstract

Preterm birth (PTB) refers to delivery before 37 weeks of gestation. Premature neonates exhibit higher neonatal morbidity and mortality rates than term neonates; therefore, predicting and preventing PTB are important. In this study, we investigated the potential of using short-chain fatty acid (SCFA) levels, specific vaginal microbiota-derived metabolites, as a biomarker in predicting PTB using gas chromatography/mass spectrometry. Cervicovaginal fluid (CVF) was collected from 89 pregnant women (29 cases of PTB vs. 60 controls) without evidence of other clinical infections, and SCFA levels were measured. Furthermore, the PTB group was divided into two subgroups based on birth timing after CVF sampling: delivery ≤ 2 days after sampling (*n* = 10) and ≥2 days after sampling (*n* = 19). The concentrations of propionic acid, isobutyric acid, butyric acid, valeric acid, hexanoic acid, and heptanoic acid were significantly higher in the PTB group than in the term birth (TB) group (*p* < 0.05). In particular, the concentrations of propionic acid, isobutyric acid, hexanoic acid, and heptanoic acid were continuously higher in the PTB group than in the TB group (*p* < 0.05). In the delivery ≤ 2 days after sampling group, the propionic acid, isobutyric acid, hexanoic acid, and heptanoic acid levels were significantly higher than those in the other groups (*p* < 0.05). This study demonstrated a significant association between specific SCFAs and PTB. We propose these SCFAs as potential biomarkers for the prediction of PTB.

## 1. Introduction

Preterm birth (PTB) refers to delivery before 37 weeks of gestation, which accounts for approximately 4–16% of babies born in 2020 across countries [1]. It is the leading cause of neonatal morbidity and mortality due to incomplete development of several organ systems [2]. Therefore, there are many efforts to predict and prevent preterm birth. The causes of PTB are very diverse with approximately two-thirds occurring after spontaneous preterm labor (PTL) without an identifiable cause and the other one-third being associated with preterm premature rupture of membranes (PPROM) [3]. The methods for predicting the risk of PTB currently used in clinical practice are history taking of previous PTB, short cervical length (<25 mm) on ultrasound in the second trimester of gestation, and a fetal fibronectin (fFN) test in cervicovaginal fluid (CVF) of pregnant women [2,4]. However, these tests have limitations, particularly their low positive predictive values [4]. Thus, the identification of new biomarkers that reliably predict PTB is required.

A high estrogen state during pregnancy causes maturation and proliferation of vaginal epithelial cells, stimulates glycogen accumulation, increases lactic acid production, and maintains a state of eubiosis that maintains a low pH environment in the vagina [5,6]. This is a condition in which *Lactobacillus* predominates and species richness is reduced [5]. However, when this balance is disrupted and the vaginal microenvironment becomes dysbiosis during pregnancy, the originally abundant *Lactobacillus* is depleted, and a series of processes occur that increase the diversity of microorganisms in the vagina [5,6,7]. Thus, lactic acid production is reduced, the vaginal pH is higher than 4.5, and the short-chain fatty acids (SCFAs) produced by anaerobic bacteria create a proinflammatory environment. Furthermore, it stimulates the secretion of proinflammatory cytokines and chemokines, prostaglandin, and matrix metalloproteinases (MMPs) and causes PTL [5,8,9,10]. These series of processes can cause intrauterine infection, and the severity varies from mild to severe enough to induce PTB [6].

With the recent development of microbiome analysis technology, research on the microbiome of pregnant women is also actively underway. Metabolomics is making substantial contributions to various research areas, such as disease diagnosis and biomarker detection [11]. Nuclear magnetic resonance (NMR) spectrometry or mass spectrometry (MS) is used to measure these metabolites. The vaginal microbiome profile of pregnant women has already been established through several studies, and the risk of PTB related to changes in the diversity and composition of the microenvironment continues to be proven [12,13,14]. Among them, SCFAs related to bacterial vaginosis could be important, and several studies have shown that SCFAs, such as acetic acid, butyric acid, succinic acid, and propionic acid, were associated with anaerobic bacteria and were detected at higher levels in the PTB group than in the term birth (TB) group [15,16].

Thus, we hypothesized that SCFAs in CVF would be higher in the PTB group, and this study investigated the potential of using SCFA levels, specific vaginal microbiota-derived metabolites, as a biomarker in PTB using gas chromatography/MS (GC–MS).

## 2. Results

### 2.1. Baseline Characteristics of the Study Participants

Table 1 shows the demographic and clinical characteristics of the 89 pregnant women (PTB group, *n* = 29; TB group, *n* = 60) and their neonates. The mean maternal age was 33.1 years in the TB group versus 31.8 years in the PTB group with no significant difference between the two groups. Furthermore, no significant differences in pre-pregnancy body mass index (BMI), nulliparity, and history of prior PTB were observed between the two groups. In addition, previous surgical history was 20% in the TB group vs. 48.3% in the PTB group with a significant difference between the two groups (*p* = 0.006). In addition, no significant differences in intrauterine injection (IUI) or in vitro fertilization–embryo transfer (IVF–ET) cases and smoking. However, clinical parameters, including cervical length, fetal fibronectin, white blood cell count, C-reactive protein, and interleukin (IL)-6 levels, were significantly different between the two groups (*p* < 0.05). No significant difference in cytokine IL-1β was observed between the two groups (*p* = 0.07). Furthermore, we divided the PTB group into two subgroups based on birth timing after CVF sampling: delivery ≤2 days after sampling (*n* = 10) and ≥2 days after sampling (*n* = 19) (Table S1). Similarly, no significant differences in the maternal age, pre-pregnancy BMI, nulliparity, and history of prior PTB were observed between the three groups. However, fetal fibronectin, white blood cell count, and C-reactive protein levels were significantly different between the three groups (*p* < 0.05). No significant difference in cervical length was observed between the three groups (Table S1).

### 2.2. Concentration of SCFAs in CVF

**Figure 1:**
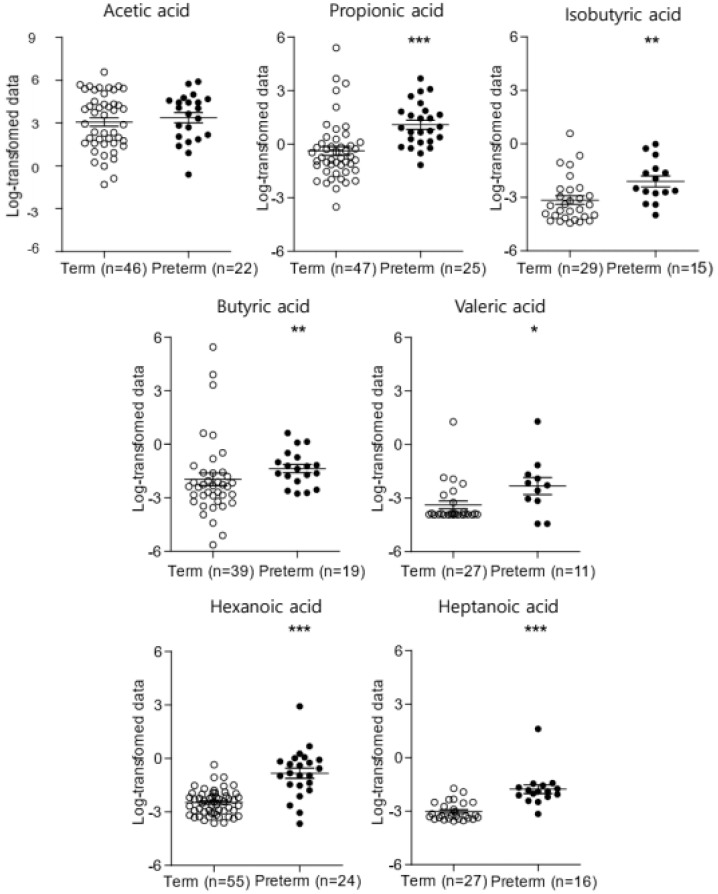
The concentration of SCFAs in CVF samples between the PTB and TB groups (* *p* < 0.05, ** *p* < 0.01, *** *p* < 0.001). SCFAs, short-chain fatty acids; CVF, cervicovaginal fluid; PTB, preterm birth; TB, term birth.

Figure 1 compares the SCFA concentrations of the PTB and TB groups. The acetic acid, propionic acid, isobutyric acid, butyric acid, isovaleric acid, valeric acid, 4-methylvaleric acid, hexanoic acid, and heptanoic acid concentrations in the CVF samples of the pregnant women were analyzed using GC–MS (Table S2). Isovaleric acid and 4-methyl valeric acid were excluded because of their low detection rate (<30%) in CVF samples. Thus, the acetic acid, propionic acid, isobutyric acid, butyric acid, valeric acid, hexanoic acid, and heptanoic acid concentrations were used to compare the two groups (Figure 1). Among them, the concentrations of propionic acid, isobutyric acid, butyric acid, valeric acid, hexanoic acid, and heptanoic acid were significantly higher in the PTB group than in the TB group (*p* < 0.05).

**Figure 2:**
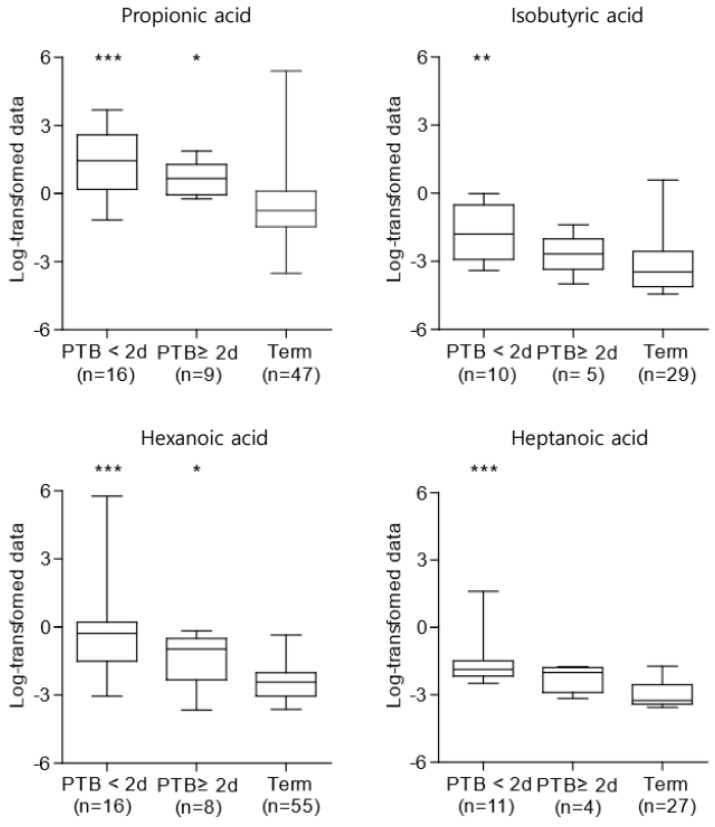
Concentrations of SCFAs in CVF samples among the PTB subgroups (* *p* < 0.05, ** *p* < 0.01, *** *p* < 0.001). They were increased in the PTB groups, particularly in the case of PTB ≤ 2 days after sampling. The Kruskal–Wallis test was used for statistical analysis. PTB, preterm birth; SCFAs, short-chain fatty acids; CVF, cervicovaginal fluid.

In particular, the concentrations of propionic acid, isobutyric acid, hexanoic acid, and heptanoic acid were persistently higher throughout gestation in the PTB group than in the TB group (*p* < 0.05) (Figure S1). For further analysis, we compared the propionic acid, isobutyric acid, hexanoic acid, and heptanoic acid concentrations between the PTB subgroups: delivery ≤2 days after sampling and ≥2 days after sampling (Figure 2). In the PTB ≤2 days after sampling group, the propionic acid, isobutyric acid, hexanoic acid, and heptanoic acid levels were significantly higher than those in the other groups (*p* < 0.05). In other words, in cases of immediate birth after hospitalization for PTL or PPROM, the concentrations of propionic acid, isobutyric acid, hexanoic acid, and heptanoic acid tended to be higher.

### 2.3. Association between SCFAs and Clinical Characteristics: Gestational Age at Birth, Time from Sampling to Birth, C-Reactive Protein, White Blood Cell Count, and IL-6

Table 2 shows the association between the SCFAs and clinical variables, which showed a significant difference, as shown in Table 1. Propionic acid, hexanoic acid, and heptanoic acid levels were inversely associated with gestational age at birth (r = −0.36, *p* = 0.002; r = −0.50, *p* < 0.001; r = −0.59, *p* < 0.001) and time from sampling to birth (r = −0.37, *p* = 0.001; r = −0.44, *p* < 0.001; r = −0.56, *p* < 0.001). Isobutyric acid level was also inversely associated with the time from sampling to birth (r = −0.42, *p* = 0.004), and hexanoic acid level was inversely associated with cervical length (r = −0.29, *p* = 0.02).

C-reactive protein level was positively correlated with propionic acid, isobutyric acid, hexanoic acid, and heptanoic acid levels (r = 0.40, *p* = 0.01; r = 0.45, *p* = 0.01; r = 0.39, *p* = 0.01; r = 0.36, *p* = 0.04). White blood cell count was positively correlated with propionic acid (r = 0.34, *p* = 0.01) and hexanoic acid (r = 0.32, *p* = 0.01) levels. Similarly, IL-6 levels were positively correlated with propionic acid (r = 0.47, *p* = 0.01), isobutyric acid (r = 0.57, *p* = 0.001), and heptanoic acid (r = 0.58, *p* = 0.002) levels (Table 2).

### 2.4. Effects of SCFA Levels on the Prediction of PTB

We used the area under the receiver operating characteristics (ROC) curve (AUC) for the SCFAs and found that propionic acid, isobutyric acid, hexanoic acid, and heptanoic acid levels were significantly associated with the prediction of PTB (<37 weeks of gestation, particularly <34 weeks of gestation) (Table 3). In the case of predicting PTB at less than 37 weeks of gestation, the sensitivity of propionic acid, isobutyric acid, hexanoic acid, and heptanoic acid was 92%, 80%, 79.2%, and 93.8%, respectively. The AUC values for these SCFAs were 0.80, 0.76, 0.87, and 0.93, respectively. Furthermore, in the case of predicting early PTB at less than 34 weeks of gestation, the sensitivity of propionic acid, isobutyric acid, and hexanoic acid was 66.7%, 100%, and 100%, respectively, and the AUC values were 0.82, 0.86, and 0.94, respectively.

## 3. Discussion

We represented the possibility of propionic acid, isobutyric acid, hexanoic acid, and heptanoic acid as markers in CVF using the GC–MS method for the PTB vs. TB group in clinical practice and found that they were associated with indicators of other inflammatory responses and were related to PTB. In particular, immediate birth within 2 days after the onset of symptoms, such as PTL, was significantly associated with higher concentrations of the SCFAs.

We focused on SCFA analysis because SCFAs are metabolites representing bacterial vaginosis, such as *Fusobacterium* and *Prevotella* [6,8]. SCFAs are the main metabolic products of anaerobic bacteria fermentation in the vagina or intestine [5,6]. It is a postbiotic metabolite and, similar to microbial metabolites produced by pathogenic bacteria such as lipopolysaccharides, exhibits pathogen-associated molecular patterns [2,17,18]. In fact, changes in vaginal bacterial community composition are associated with changes in metabolite characteristics and with an increase in intrauterine infections, leading to spontaneous PTL [4]. This is a plausible explanation for the association between a higher prevalence of mixed anaerobes (e.g., *Gardnerella*, *Fusobacterium*, *Bacteroides*, *Mobiluncus*, and *Mycoplasma*) and PTB [8]. If bacterial vaginosis becomes an ascending infection during pregnancy, it can cause serious obstetric complications, such as PPROM, PTL, and PTB, and in the case of chorioamnionitis, it also affects the prognosis of the neonates [19]. In this way, SCFAs can serve as an intermediate substance that can predict the risk of PTB due to vaginal dysbiosis. In particular, high propionic acid, isobutyric acid, hexanoic acid, and heptanoic acid levels were always observed in the PTB group after the second trimester of gestation, indicating that the risk of PTB can be predicted regardless of the time of sample collection. Furthermore, the predictive performance of propionic acid, isobutyric acid, and hexanoic acid for early PTB before 34 weeks of gestation was significant, which may be notable results in determining the prognosis of preterm neonates and deciding the use of antenatal steroids.

Furthermore, intrauterine infection through ascending infection during pregnancy stimulates the secretion of proinflammatory cytokines and chemokines, prostaglandins, and MMPs in the mother and corticotropin-releasing hormone in the fetus [2]. These cause PTL through a series of processes, including uterine contraction, cervix remodeling, and membrane activation [2,6]. IL-6 is a highly sensitive and specific indicator of infection-induced PTL [12,19,20]. Furthermore, several studies have shown that elevated IL-6, IL-1α, and IL-β levels in amniotic fluid and IL-6 and IL-17α levels in CVF are correlated with PTB [21,22,23]. According to the aforementioned findings, increased levels of inflammatory cytokines in CVF are related to PTB. As shown in Table 2, propionic acid, isobutyric acid, and heptanoic acid were correlated with IL-6; therefore, SCFAs can be developed as predictive markers of PTB, similar to inflammatory cytokines.

Usually, in clinical practice, vaginal infection is diagnosed through symptoms, pH, polymerase chain reaction, or culture in CVF. In this study, specific SCFAs, which can be biomarkers for PTB, were validated using GC–MS. Usually, NMR spectrometry and MS are used to measure metabolites in metabolomics. They are inherently complementary with distinct strengths and limitations [24,25]. In this study, it was finally represented through GC–MS that propionic acid, isobutyric acid, hexanoic acid, and heptanoic acid were potential markers that could predict the risk of PTB. In fact, in the case of acetic acid, which was not different between the PTB and TB groups, GC–MS might not be an accurate method for analysis because of its volatile nature. In our previous study, acetone, ethanol, ethylene glycol, formic acid, glycolate, isopropanol, methanol, and trimethylamine N-oxide were significantly increased in the PTB group (particularly in the case of birth within 7 days after CVF sampling) compared with those in the TB group by NMR spectrometry [15]. However, NMR spectrometry is expensive, and there are few places where it can be used. In fact, the risk of PTB caused by dysbiosis has limitations that make it difficult to accurately explain it with a single component. Therefore, both GC–MS and NMR spectrometry should be used to supplement the detection of metabolites related to PTB and, thereby, understand the metabolic process [26]. However, in metabolomics, metabolites are mainly detected by GC–MS because of the possibility of rapid and cost-effective analysis [24]. This feature is essential for biomarker measurements to be used in clinical practice.

Our study has some limitations. Because this study had a relatively small sample size, the results must be validated through a larger study. Second, the independence of inflammatory indicators and SCFAs was not confirmed in the correlation between SCFAs and PTB.

In this study, propionic acid, isobutyric acid, hexanoic acid, and heptanoic acid, which were highly measured in the CVF of the PTB group, can be used as indirect biomarkers for predicting the risk of PTB. Combining SCFAs with inflammatory cytokine levels (e.g., IL-6) or other clinically used methods for predicting PTB (e.g., fFN) or the concentration of retinoid metabolites shown in another study could become a powerful tool for predicting PTB [27,28]. As mentioned above, understanding the metabolite changes in pregnant women will not only help us understand the mechanism of PTB caused by inflammation but may also uncover potential new treatments.

## 4. Materials and Methods

### 4.1. Study Design and Participants

**Figure 3:**
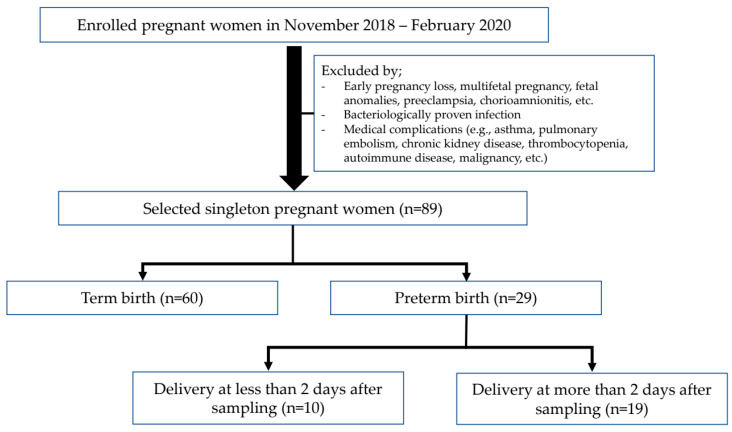
Study flowchart for participants composition.

This nested case–control study was conducted on pregnant women who delivered at Ewha Womans University Mokdong Hospital from 1 November 2018 to 29 February 2020. The 89 participants were divided into two groups: 29 cases of PTB and 60 controls. Furthermore, the PTB group was divided into two subgroups based on birth timing after CVF sampling: delivery ≤2 days after sampling (*n* = 10) and ≥2 days after sampling (*n* = 19) (Figure 3). Women with obstetric pregnancy complications (e.g., early pregnancy loss, multifetal pregnancy, fetal anomalies, preeclampsia, chorioamnionitis) and non-obstetric complications (e.g., bacteriologically proven infection, asthma, pulmonary embolism, chronic kidney disease, thrombocytopenia, autoimmune disease, malignancy, etc.) were excluded. This study’s protocol was approved by the Institutional Review Board (IRB) of the Ewha Womans University Mokdong Hospital (IRB No. EUMC 2018-07-007). Written informed consent was obtained from all participants at enrollment, and the consent procedure was approved by the IRB.

PTB was defined as delivery at <37 weeks of gestation, based on the last menstrual period confirmed or modified by ultrasound evaluation [29]. Spontaneous PTL was defined as the presence of intact membranes and regular contractions, whereas PPROM was defined as the rupture of membranes before the onset of PTL [2]. Data on the clinical, demographic, and socioeconomic variables of the participants with or without PTB were obtained from obstetric and neonatal medical records.

### 4.2. CVF Collection and Preparation

CVF samples from the participants were collected from the posterior fornix of the cervix using sterile cotton-tipped swabs at the second or third trimester of gestation. CVF samples were collected in 0.5 mL phosphate-buffered saline and stored at −80 °C. They were not thawed until analysis.

**Figure 4:**
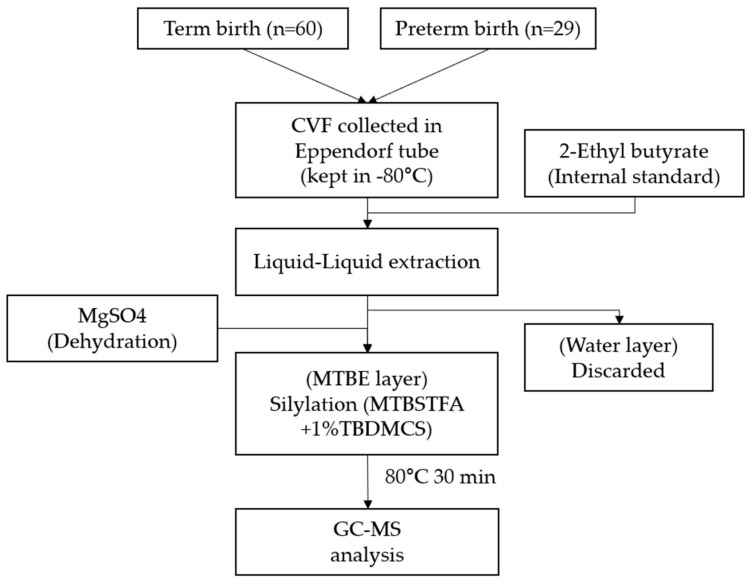
Technical process of CVF preparation to GC–MS analysis. CVF, cervicovaginal fluid; MTBE, *tert-*butyl methyl ether (MTBE); MTBSTFA, *N-tert-*butyldimethylsilyl-*N*-methyltrifluoroacetamide; TBDMCS, *tert-*butyldimethylchlorosilane.

For the SCFA extraction process in CVF, 50 μL of sample was placed in a 0.5 mL microtube, and 40 μL of 2-ethyl butyrate (internal standard, 1 μg/mL) dissolved in *tert-*butyl methyl ether (MTBE) was added. The mixture was vortexed for 30 s and centrifuged (2200× *g*, 4 °C) for 5 min. The supernatant (organic layer) was transferred to another tube containing 15 mg of MgSO_4_, and the extraction process was repeated twice using MTBE as the extraction solvent. It was then transferred to a 2 mL vial, and the collected extract was inserted. Ten microliters of *N*-*tert*-butyldimethylsilyl-*N*-methyltrifluoroacetamide (MTBSTFA) along with 1% *tert-*butyldimethylchlorosilane reagent was added to the vial, derivatized at 80 °C for 30 min, and then cooled at room temperature for 20 min. The final solution was then injected into the GC–MS instrument (Figure 4) [30].

### 4.3. Metabolomic and Cytokine Analyses

GC was performed on an Agilent 7890B GC system from Agilent Technologies (Santa Clara, CA, USA), and MS was performed on an Agilent 5877A MSD from Agilent Technologies. HP-5MS UI (30 m × 0.25 mm, 0.25 μm) was used for chromatographic separation [30].

**Figure 5:**
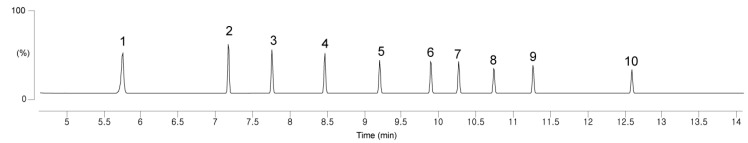
GC–MS chromatogram of blank sample spiked with SCFAs and an internal standard. (1) acetic acid, (2) propionic acid, (3) isobutyric acid, (4) butyric acid, (5) isovaleric acid, (6) valeric acid, (7) 2-ethyl butyric acid (internal standard), (8) 4-methyl valeric acid, (9) hexanoic acid, (10) heptanoic acid.

One microliter of derivatized SCFAs was injected into the GC–MS with a split ratio of 5:1. The carrier gas was helium with a flow rate of 1 mL/min. The initial GC oven temperature setting was 50 °C (maintained for 2 min), increased to 150 °C at a rate of 10 °C/min, and increased to 310 °C at a rate of 15 °C/min maintained for 12.4 min with a total run time of 35 min. The other GC–MS parameters were set as follows: inject temperature, 240 °C; interface temperature, 300 °C; ion source, 240 °C; and quadrupole, 150 °C. Ionization was performed in the electron impact mode at 70 eV [31]. The selected ion monitoring mode was set with the target ions (*m*/*z*) of acetic acid (117), propionic acid (131), isobutyric acid (145), butyric acid (145), isovaleric acid (159), valeric acid (159), 4-methylvaleric acid (173), hexanoic acid (173), and heptanoic acid (189) as quantitative ions (Figure 5) [30].

Quantitative IL-6 and IL-1β levels were measured using the human IL-6 and IL-1β enzyme-linked immunosorbent kit (Abbkine, Wuhan, China) according to the manufacturer’s instructions.

### 4.4. Data Processing and Statistical Analysis

Data analysis was performed using MassHunter Workstation GC–MS Data Acquisition (ver. 07.00), Agilent MassHunter Qualitative Analysis (ver. 10.0), Agilent MassHunter Quantitative Analysis (ver. 10.1), Quantitative Analysis Library Editor (ver. 10.1), and NIST Mass Spectral Search Program (ver. 2.3) [30].

Clinical characteristics were compared using Student’s *t*-test for continuous variables. The Mann–Whitney U test was used to compare SCFA levels in CVF between the two groups. Moreover, we performed the Kruskal–Wallis test with Tukey’s post hoc test to compare the characteristics of the PTB group (delivery at less than 2 days after sampling against delivery at more than 2 days after sampling vs. TB). The concentrations of SCFAs were log-transformed to satisfy normality. We measured the association between SCFAs and clinical variables using Spearman’s rank correlation. The predictive potential of SCFAs for PTB was determined by applying the AUC of the ROC curves.

Statistical analysis was performed using SAS (ver. 9.4; SAS Institute Inc., Cary, NC, USA). All analyses were two-tailed, and *p*-values of <0.05 were used to denote statistical significance.

### 4.5. Method Validation

Method validation was performed to prove the reliability of the developed method. Based on “Guidance for Industry: Bioanalytical Method Validation” of the U.S. Food and Drug Administration (FDA) on bioanalytical method validation, method validation was performed on accuracy, precision, linearity, selectivity, and lower limit of quantification following a previous study [30,32].

## 5. Conclusions

This study demonstrated a significant association between SCFAs (i.e., propionic acid, isobutyric acid, hexanoic acid, and heptanoic acid) and PTB. These metabolites are associated with inflammatory responses that stimulate the pathway to PTB and can be predictive markers of PTB. If we combine propionic acid, isobutyric acid, hexanoic acid, and heptanoic acid with inflammatory cytokine levels or other clinically used methods for predicting PTB, it could become a powerful tool for predicting PTB [27,28]. In addition, The GC–MS method to detect the SCFAs can be a rapid and cost-effective analysis in a clinical setting.

## Figures and Tables

**Table 1:** Clinical characteristics of the study participants.

	TB (*n* = 60)	PTB (*n* = 29)	
	Mean ± SD or N (%)	Mean ± SD or N (%)	*p*
Maternal age (years)	33.1 ± 3.9	31.8 ± 4.1	0.14
Pre-pregnancy BMI (kg/m^2^)	21.4 ± 2.5	22.3 ± 3.1	0.16
Nulliparity	39 (65.0)	14 (51.9)	0.25
History of prior PTB	2 (3.3)	3 (10.3)	0.18
History of previous surgery	12 (20.0)	15 (48.3)	0.006
ART (IUI or IVF–ET)	2 (3.3)	2 (6.8)	0.351
Smoking	0	0	-
Cervical length (mm)	29.1 ± 7.2	24.0 ± 8.9	0.01
Fetal fibronectin			
Positive	1 (16.7)	12 (80.0)	0.01
Negative	5 (83.3)	3 (20.0)	
White blood cells (cell/mL)	8.8 ± 2.1	10.6 ± 3.0	0.01
C-reactive protein (mg/L)	0.2 ± 0.2	0.8 ± 1.1	0.01
IL-6 (pg/mL)	3.5 ± 4.2	35.9 ± 60.5	0.003
IL-1β (pg/mL)	76.7 ± 79.8	119.5 ± 115.9	0.07
Gestational age at birth (weeks)	38.7 ± 0.9	32.9 ± 3.8	<0.001
Sex of the neonates			
Male	34 (57.6)	16 (59.3)	0.89
Female	25 (42.4)	11 (40.7)	
Birth weight (g)	3143.0 ± 308.7	2193.1 ± 623.2	<0.001
Apgar score (1 min)	9.5 ± 1.5	6.7 ± 3.0	<0.001
Apgar score (5 min)	9.7 ± 1.4	8.4 ± 2.7	0.02

Continuous data are presented as means ± standard deviations, and categorical data are presented as N (%). TB, term birth; PTB, preterm birth; BMI, body mass index; ART, artificial reproductive technologies; IUI, intrauterine injection; IVF–ET, in vitro fertilization–embryo transfer.

**Table 2:** Correlation between SCFAs and clinical characteristics.

	Propionic Acid	Isobutyric Acid	Hexanoic Acid	Heptanoic Acid
Gestational age at birth	−0.36 (0.002)	−0.30 (0.05)	−0.50 (<0.001)	−0.59 (<0.001)
Time from sampling to birth	−0.37 (0.001)	−0.42 (0.004)	−0.44 (<0.001)	−0.56 (<0.001)
Cervical length	−0.20 (0.13)	−0.08 (0.63)	−0.29 (0.02)	−0.10 (0.53)
C-reactive protein	0.40 (0.01)	0.45 (0.01)	0.39 (0.01)	0.36 (0.04)
White blood cell count	0.34 (0.01)	0.25 (0.16)	0.32 (0.01)	0.24 (0.17)
IL-6	0.47 (0.001)	0.57 (0.001)	0.21 (0.15)	0.58 (0.002)

Values are represented as Spearman’s correlation coefficients (r) and *p*-values. SCFAs, short-chain fatty acids.

**Table 3:** Predictive performance of PTB on SCFA levels in CVF.

	GA < 37 Weeks				GA < 34 Weeks			
	OR (95% CI)	AUC (95% CI)	Sensitivity (%)	Specificity (%)	OR (95% CI)	AUC (95% CI)	Sensitivity (%)	Specificity (%)
Propionic acid	1.80 (1.25, 2.58) *	0.80 (0.70, 0.91) *	92.0	63.8	1.93 (1.29, 2.89) *	0.82 (0.71, 0.94) *	66.7	91.2
Isobutyric acid	1.90 (1.11, 3.25) *	0.76 (0.62, 0.91) *	80.0	72.4	2.63 (1.37, 5.04) *	0.86 (0.75, 0.97) *	100	72.7
Hexanoic acid	5.61 (2.59, 12.19) *	0.87 (0.77, 0.98) *	79.2	90.9	6.15 (2.54, 14.91) *	0.94 (0.89, 0.99) *	100	79.7
Heptanoic acid	28.01 (4.27, 183.49) *	0.93 (0.85, 1.00) *	93.8	85.2	44.61 (3.38, 588.82) *	0.93 (0.85, 1.00)	90.9	84.4

The predictive potential of the SCFAs for PTB was determined by applying the area under the receiver operating characteristic curves (* *p* < 0.05).

## Data Availability

The data presented in this study are available on request from the corresponding author. The data are not publicly available due to ethical reasons.

## Supplementary Materials

The following supporting information can be downloaded at: https://www.mdpi.com/article/10.3390/ijms25063396/s1.
