# Supplementary material for: Identification of Short-Chain Fatty Acids for Predicting Preterm Birth in Cervicovaginal Fluid Using Mass Spectrometry
Source: Int J Mol Sci. 2024 Mar 17;25(6):3396. doi: 10.3390/ijms25063396 (PMC10970234; doi:10.3390/ijms25063396)
Supplement: Supplementary file 1 [file ijms-25-03396-s001.zip › ijms-2883522-supplementary.pdf]

Article

Supplementary Materials: Identification of Short-Chain Fatty Acids for Predicting Preterm Birth in Cervicovaginal Fluid Using Mass Spectrometry

Young-Min Hur [1,†], Eun-Jin Kwon [2,†], Young-Ah You [1], Sunwha Park [1], Soo-Min Kim [1], Gain Lee [1], Yoon-Young Go [1] and Young-Ju Kim [1,*]

[1] Department of Obstetrics and Gynecology, Ewha Medical Research Institute, College of Medicine, Ewha Womans University, Seoul 07984, Republic of Korea; hym1210@ewha.ac.kr (Y.-M.H.); yayou@ewha.ac.kr (Y.-A.Y.); clarrissa15@gmail.com (S.P.); zeus_0218@ewhain.net (S.-M.K.); loveleee0102@gmail.com (G.L.); gokogoko@ewha.ac.kr (Y.-Y.G.)

[2] Division of Allergy and Respiratory Disease Research, Department of Chronic Disease Convergence Research, Korea National Institute of Health, Cheongju-si 28159, Republic of Korea; friendkej1004@hanmail.net

* Correspondence: kkyj@ewha.ac.kr

† These authors contributed equally to this work.

Table S1. Clinical characteristics of the subgroup of PTB

	TB (n=60)	PTB < 2 days (n=19)	PTB ≥ 2 days (n=10)	
	**Mean ± SD or N (%)**	**Mean ± SD or N (%)**	**Mean ± SD or N (%)**	***P***
Maternal age (years)	33.1 ± 3.9	31.3 ± 4.0	32.6 ± 4.4	0.23
Pre-pregnancy BMI (kg/m^2)	21.4 ± 2.5	22.5 ± 3.2	21.8 ± 2.8	0.18
Nulliparity	39 (65.0)	11 (57.9)	3 (37.5)	0.31
History of prior PTB	2 (3.3)	2 (10.5)	1 (10.0)	0.40
Cervical length (mm)	29.1 ± 7.2	22.5 ± 9.0	26.6 ± 8.4	0.09
Fetal fibronectin				
Positive	1 (16.7)	11 (91.7)	1 (33.3)	0.01
Negative	5 (83.3)	1 (8.3)	2 (66.7)	
White blood cell (cell/mL)	8.8 ± 2.1	10.8 ± 2.9	10.2 ± 3.3	0.03
C-reactive protein (mg/L)	0.2 ± 0.2	0.9 ± 1.3	0.8 ± 0.9	0.003
Gestational age at birth (weeks)	38.7 ± 0.9	32.3 ± 3.8	34.0 ± 3.5	<0.001

Kruskal–Wallis test was used for the statistical analysis. Continuous data are presented as means ± standard deviations, and categorical data are presented as N (%).
TB, term birth; PTB, preterm birth; BMI, body mass index.

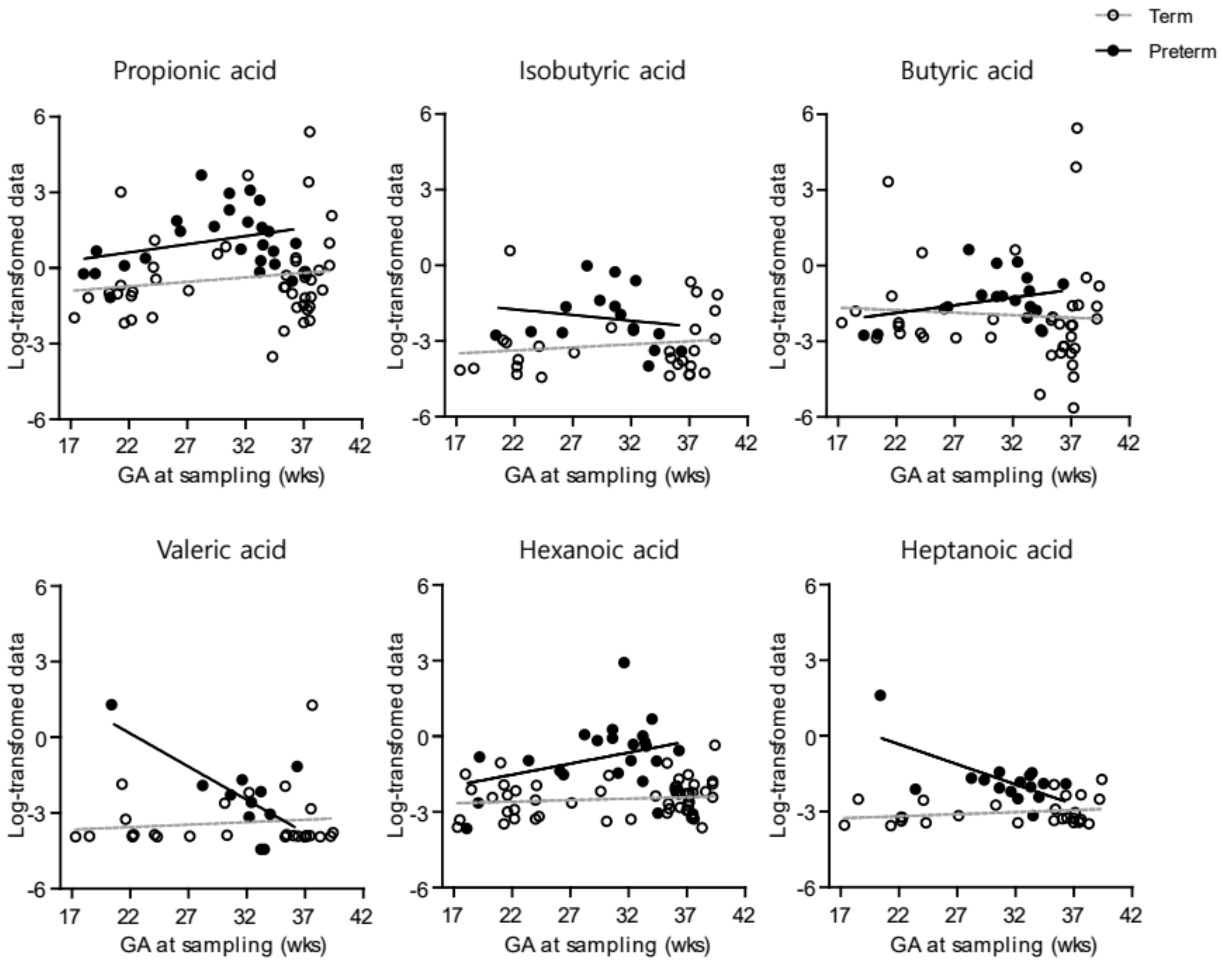

Figure S1. Concentration of SCFAs in CVF samples from second to third trimester of pregnancy

GA, gestational age; SCFAs, short chain fatty acids; CVF, cervicovaginal fluid

Table S2. Quantitative results of SCFAs in clinical sample

(Unit: μg/mL)

Patient No.	Acetic acid	Propionic acid	Isobutyric acid	Butyric acid	Valeric acid	Hexanoic acid	Heptanoic acid
**1**	N.D.	0.883	N.D.	0.143	BLOQ	0.132	N.D.
**2**	N.D.	0.895	N.D.	N.D.	N.D.	0.149	N.D.
**3**	N.D.	0.836	N.D.	0.047	N.D.	0.164	N.D.
**4**	3.003	0.767	N.D.	0.065	N.D.	0.162	N.D.
**5**	N.D.	0.819	N.D.	BLOQ	BLOQ	0.136	N.D.
**6**	20.037	0.223	0.049	0.193	0.065	0.206	0.104
**7**	1.64	N.D.	N.D.	0.091	N.D.	0.255	N.D.
**8**	N.D.	1.316	N.D.	0.108	N.D.	0.219	N.D.
**9**	40.196	10.585	0.096	14.971	0.067	0.106	0.095
**10**	42.765	0.452	0.481	0.34	2.405	0.103	0.101
**11**	N.D.	N.D.	N.D.	N.D.	N.D.	0.187	N.D.
**12**	18.737	0.256	0.056	0.208	0.065	0.082	0.086
**13**	0.538	0.489	0.128	N.D.	N.D.	0.481	N.D.
**14**	N.D.	N.D.	N.D.	N.D.	N.D.	N.D.	N.D.
**15**	33.235	0.239	0.05	0.188	0.065	0.133	0.097
**16**	4.997	0.5	N.D.	0.136	N.D.	0.185	N.D.
**17**	0.404	0.177	0.048	0.085	0.065	0.121	0.098
**18**	42.006	0.442	0.059	0.286	0.066	0.231	0.175
**19**	95.002	42.284	0.172	43.828	0.141	0.112	0.094
**20**	13.229	0.316	N.D.	0.103	N.D.	N.D.	N.D.
**21**	18.016	2.15	N.D.	1.42	N.D.	N.D.	N.D.
**22**	19.84	0.94	0.052	0.716	0.065	0.081	0.089
**23**	N.D.	0.236	N.D.	N.D.	N.D.	0.211	N.D.
**24**	47.404	0.463	0.062	0.207	0.068	0.104	0.107
**25**	N.D.	N.D.	N.D.	N.D.	N.D.	0.208	N.D.
**26**	4.898	N.D.	N.D.	N.D.	N.D.	0.353	N.D.
**27**	17.361	N.D.	N.D.	N.D.	N.D.	N.D.	N.D.
**28**	44.25	0.591	0.094	0.223	0.26	0.475	0.263
**29**	11.815	0.906	0.632	0.33	N.D.	0.349	N.D.
**30**	27.207	1.107	N.D.	0.164	N.D.	0.121	N.D.
**31**	21.537	0.897	N.D.	0.238	0.046	0.289	N.D.
**32**	N.D.	1.595	N.D.	0.148	BLOQ	0.566	N.D.
**33**	1.871	0.86	N.D.	BLOQ	BLOQ	0.16	N.D.
**34**	N.D.	1.668	BLOQ	BLOQ	0.311	7.551	0.217
**35**	0.653	0.852	N.D.	BLOQ	N.D.	0.079	N.D.
**36**	23.914	6.387	BLOQ	0.712	0.225	1.007	0.34

**37**	2.617	2.717	0.097	0.292	0.121	1.601	0.185
**38**	4.143	3.533	0.177	0.384	0.112	0.512	0.178
**39**	N.D.	1.885	0.063	0.324	0.046	0.758	0.112
**40**	31.742	8.438	0.655	1.1	0.167	0.794	0.281
**41**	N.D.	N.D.	N.D.	N.D.	N.D.	N.D.	N.D.
**42**	53.543	12.777	0.989	1.548	0.266	1.042	0.313
**43**	12.472	N.D.	0.259	0.431	N.D.	0.363	N.D.
**44**	N.D.	0.444	0.147	0.152	2.449	54.362	3.055
**45**	N.D.	N.D.	N.D.	N.D.	N.D.	N.D.	N.D.
**46**	N.D.	N.D.	N.D.	N.D.	N.D.	N.D.	N.D.
**47**	7.061	1.968	0.095	0.601	0.448	0.673	0.268
**48**	6.129	1.326	N.D.	0.084	0.125	0.426	0.191
**49**	21.753	4.934	0.322	0.427	0.204	0.946	0.239
**50**	9.961	1.581	0.153	0.171	N.D.	0.502	0.269
**51**	16.629	2.731	0.318	0.321	N.D.	0.348	N.D.
**52**	3.172	0.698	N.D.	N.D.	N.D.	0.23	N.D.
**53**	4.515	1.21	N.D.	N.D.	N.D.	N.D.	0.246
**54**	21.768	3.062	BLOQ	0.495	N.D.	0.849	0.363
**55**	6.5	1.312	0.161	N.D.	N.D.	0.512	0.231
**56**	25.606	3.135	0.382	0.445	N.D.	0.891	0.298
**57**	59.134	7.808	0.835	1.067	N.D.	1.198	0.371
**58**	3.637	1.063	N.D.	N.D.	N.D.	N.D.	N.D.
**59**	16.32	3.666	0.157	0.303	N.D.	0.387	N.D.
**60**	5.158	N.D.	N.D.	N.D.	N.D.	N.D.	N.D.
**61**	1.886	N.D.	N.D.	0.02	N.D.	0.207	N.D.
**62**	22.74	0.437	0.063	0.194	0.067	0.131	0.122
**63**	2.864	0.087	N.D.	0.029	N.D.	0.194	N.D.
**64**	44.354	0.594	0.078	0.243	0.069	0.157	0.133
**65**	47.477	0.492	0.066	0.2	0.067	0.249	0.103
**66**	3.002	1.205	N.D.	N.D.	N.D.	0.308	0.195
**67**	15.514	0.257	N.D.	N.D.	N.D.	0.173	N.D.
**68**	N.D.	0.618	N.D.	N.D.	N.D.	0.263	N.D.
**69**	40.031	0.516	0.075	0.155	0.069	0.224	0.105
**70**	3.926	N.D.	N.D.	N.D.	N.D.	0.205	N.D.
**71**	7.608	1.476	N.D.	N.D.	N.D.	0.221	N.D.
**72**	15.911	8.056	0.119	10.01	0.276	0.09	0.085
**73**	3.656	N.D.	N.D.	N.D.	N.D.	0.125	N.D.
**74**	2.992	0.717	N.D.	N.D.	N.D.	0.265	0.197
**75**	1.388	N.D.	N.D.	0.14	0.164	0.097	N.D.
**76**	3.783	1.021	0.108	0.156	0.069	0.259	0.171

**77**	N.D.	N.D.	N.D.	N.D.	N.D.	0.101	N.D.
**78**	16.835	1.986	0.288	0.325	N.D.	0.269	N.D.
**79**	N.D.	0.346	N.D.	N.D.	N.D.	0.118	N.D.
**80**	N.D.	BLOQ	N.D.	N.D.	N.D.	0.103	N.D.
**81**	3.37	0.731	0.046	0.141	0.065	0.11	0.092
**82**	3.844	1.075	0.132	0.232	0.066	0.289	0.176
**83**	1.989	0.538	0.091	0.137	0.066	0.16	0.112
**84**	14.877	4.211	0.444	0.571	0.073	0.781	0.302
**85**	51.123	12.697	0.167	1.535	0.219	0.102	0.092
**86**	N.D.	0.547	N.D.	N.D.	N.D.	0.219	N.D.
**87**	7.338	1.787	0.181	0.229	0.068	0.342	0.15
**88**	0.982	0.364	0.05	0.09	0.065	0.129	0.093
**89**	1.178	0.34	0.073	0.109	0.066	0.142	0.105
